# Simple, Time-Saving Dye Staining of Proteins for Sodium Dodecyl Sulfate–Polyacrylamide Gel Electrophoresis Using Coomassie Blue

**DOI:** 10.1371/journal.pone.0022394

**Published:** 2011-08-05

**Authors:** Wei-Hua Dong, Tian-Yun Wang, Fang Wang, Jun-He Zhang

**Affiliations:** Department of Biochemistry and Molecular Biology, Xinxiang Medical University, Xinxiang, Henan, China; University of South Florida, United States of America

## Abstract

A fixation-free and fast protein-staining method for sodium dodecyl sulfate–polyacrylamide gel electrophoresis using Coomassie blue is described. The protocol comprises staining and quick washing steps, which can be completed in 0.5 h. It has a sensitivity of 10 ng, comparable with that of conventional Coomassie Brilliant Blue G staining with phosphoric acid in the staining solution. In addition, the dye stain does not contain any amount of acid and methanol, such as phosphoric acid. Considering the speed, simplicity, and low cost, the dye stain may be of more practical value than other dye-based protein stains in routine proteomic research.

## Introduction

Polyacrylamide gel electrophoresis (PAGE) has become the most common method for protein analysis and detection in molecular biology experiments [1]–[4]. Subsequent to separation by electrophoresis, proteins in a gel are detected by several staining methods, such as Coomassie blue, silver, and fluorescent staining methods. [1]. Silver staining is one of the most sensitive protein-staining methods and could detect proteins at nanogram levels. However, it has the following disadvantages: difficulty in the operation and low repetition rate. Moreover, silver staining presents worse mass-spectrometry (MS) compatibility compared with the traditional Coomassie blue staining because it includes glutaraldehyde in the sensitization solution. It has also been noted as an MS-incompatible method. Fluorescent staining could detect proteins at the nanogram level without the requirement of special technical skill. At present, SYPRO orange, SYPRO red, Deep Purple, and Flamingo are frequently used in proteomics research. However, fluorescent staining is inconvenient because it requires special equipment, such as a fluorescent imaging scanner and an integrator [5]–[7].

In spite of the high sensitivity of silver staining and the wide dynamic range of various fluorescent detection methods, Coomassie Brilliant Blue (CBB) staining remains the most commonly used detection technique for proteins separated by electrophoresis [5], [7]. CBB staining was first developed to stain proteins on a cellulose acetate sheet in 1963 [8]. Subsequently, a protocol for staining proteins in polyacrylamide gels using a methanol/acetic acid/water mixture (5∶1∶5) as solvent system for CBB R-250 was described [9]–[12]. Coomassie blue staining has the following advantages: low cost, visual inspection, easy operation, convenient scanning procedure for image acquisition, better suitability for quantitative analysis than silver staining, and the capacity to allow possible modifications for fast or highly sensitive staining. Coomassie stain has become the key compound in Blue native PAGE in the last 10 years [13], [14]. However, it still requires a long staining time, and the relatively complicated ingredients make its use inconvenient. In the present study, we present an improved method for in-gel staining of proteins, which has the advantage of speed over the conventional CBB staining, yet has a comparable sensitivity.

## Materials and Methods

### Reagents

Acrylamide and bis-acrylamide were purchased from Sigma (St. Louis, MO, USA). Bovine serum albumin (BSA) and CBB R-250 were purchased from Takara Co., Ltd., Japan. All reagents used were of analytical grade.

### Preparation of staining solution

CBB R-250 (500 mg) was dissolved in 1000 ml of distilled water by stirring for 2–4 h. Subsequently, the solution was heated to 50°C for complete dissolution of CBB R-250.

### Gel electrophoresis

The stacking and separating gels used were 3.0% and 10% polyacrylamide, respectively, with an acrylamide : bis-acrylerolamide ratio of 30∶0.8. The running buffer was prepared from 25 mM Tris base, 0.2 M glycine, and 0.1% sodium dodecyl sulfate (SDS) with pH of 8.3. Concentrations of BSA were determined by the Bradford method [15]. Prior to electrophoresis, the samples were heated in the presence of sample buffer (70 mM Tris–HCl, pH 6.8, 11.4% glycerol, 3% SDS, 0.01% bromphenol blue, and 5% b-mercaptoethanol) at 100°C for 5 min in a boiling water bath [15]. Protein samples were loaded into the individual wells, and electrophoresis processed 1 mA/cm gel in the stacking gels and 2 mA/cm gel in the separating gels.

### Protein staining and image acquisition

After separation, the gels were carefully transferred to a steel tray filled with distilled water, heated to 100°C, and cooled to room temperature to remove the water. The staining solution was added into the gel, heated to 100°C, and kept at this temperature for 30–60 s. Subsequently, the stained gels were removed from the staining solution and boiled in distilled water for 30–60 s. The washing step was repeated several times. The gel images were acquired by Tocan 240 system (Tocan Biotechnology Corporation, Shanghai, China) in a UV-1 mode.

## Results and Discussion

### Sensitivity

**Figure 1:**
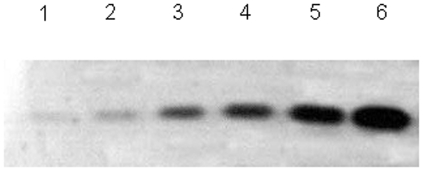
Sensitivity of the described procedure. Different concentrations of BSA were separated by SDS-PAGE and stained with water-soluble CBBR at boiling temperature for 60 s. Lanes 1–6 represent 10 ng, 100 ng, 500 ng, 1 µg, 5 µg, and 10 µg of BSA.

The Neuhoff's Chinacolloidal Coomassie Blue G-250 staining has a detection limit of approximately 10 ng of protein per spot [16]. To evaluate the sensitivity of this approach, different concentrations of BSA samples were separated on SDS-PAGE. The results showed that this approach has a sensitivity of 10 ng, which is the same as the traditional Coomassie blue stains [16] and other fast Coomassie stains [15], [17] (Fig. 1).

### Different acids

**Figure 2:**
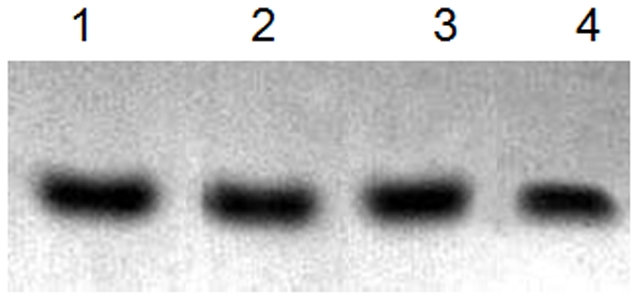
Gels stained with different staining solutions. BSA (500 ng) was separated by SDS-PAGE and stained with different acid-soluble CBBR at boiling temperature for 60 s. Added to the water-soluble CBBR staining solution were 0.3% HCl (Lane 1), 0.3% phosphoric acid (Lane 2), and 0.3% acetic acid (Lane 4), respectively.

To evaluate whether the acids have any effects on the protocol, we used different acids, including hydrochloric acid, phosphoric acid, and acetic acid, to prepare the staining solution in the experiment. No significant difference was found between staining with different acids solutions and with no acid solution. The water solution with and without acid could dye the protein just as well in this staining protocol (Fig. 2, lane 3). All the traditional staining solutions contain methanol, acetic acid, or phosphoric acid which not only produce unpleasant smell but also cause environmental pollution. In the dye stain, no acids are needed for the staining solution preparation.

### Different time and temperature

**Figure 3:**
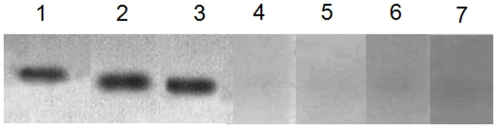
Gels stained at different time periods. BSA (500 ng) was separated by SDS-PAGE and stained with water-soluble CBBR solution at boiling temperature for 30 s (Lane 1), 60 s (Lane 2), and 120 s (Lane 3) and at room temperature for 1 h (Lane 4), 6 h (Lane 5), 14 h (Lane 6), and 24 h (Lane 7).

Temperature is a very important factor for protein detection in the protocol. We compared the gel staining under different boiling times and different staining times at room temperature. Boiling for 30–60 s was found to be enough to obtain a clear band (Fig. 3). This indicates better time-saving ability compared with conventional Coomassie [16] and other fast Coomassie staining methods [15], [17]. However, staining with this quick staining solution at room temperature was difficult, and no clear bands were acquired even after staining for 24 h (Fig. 3, lane 7). The color of the bands and backgrounds became more unclear as the staining time increased. The backgrounds were very difficult to retain (Fig. 3). Therefore, boiling temperature was necessary to obtain a clear band and clean background.

CBB R-250 binding to proteins is mainly achieved by electrostatic interaction between sulfonic (SO_3_^−^) group of dyes and protonated (NH_3_^+^) groups of proteins. Additionally, van der Waals forces and hydrogen bonding contribute to the binding interaction between dye and protein [18]. The mechanism for hot fast CBB staining with the rise of temperature is possibly because of the increasing speed of the molecules of the dye and proteins. In addition, the collision chance of the sulfonic (SO_3_^−^) group of dyes and protonated (NH_3_^+^) groups of proteins increases at boiling temperature, resulting in easier staining of the bands of proteins.

### Water purity

**Figure 4:**
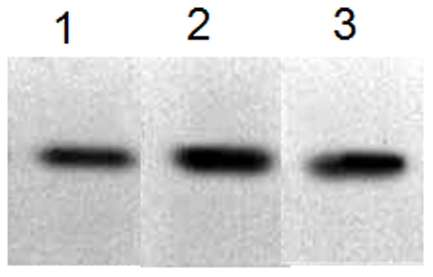
Gel washing with different types of water. BSA (500 ng) was separated by SDS-PAGE, and the separated gels were washed with different types of water: running water (Lane 1), distilled water (Lane 2), and double-distilled water (Lane 3) and stained with water-soluble CBBR solution at boiling temperature for 60 s.

Washing before staining is necessary to prepare a clean background. Different types of water were used to wash the gels. We found that water has slight effect on the staining results (Fig. 4). When the gels were washed using tap water, the bands were clear, but the sensitivity was worse than those of the bands washed with distilled water and double-distilled water (Fig. 4, lane 1). There was no significant difference between washing with distilled water and washing with double-distilled water.

In conclusion, the protocol described in the present study has four advantages: simplicity, low cost, environmental friendliness, and time-saving ability. Moreover, the protocol required only two reagents (Coomassie blue and distilled water) for the staining solution; fixing and destaining are performed only with distilled water. Traditional staining processes require many reagents, such as EtOH or MeOH, HAc, glutaraldehyde, NaAc, formalin, Na_2_CO_3_, EDTA, AgNO_3_, etc. [16]. These reagents are not only expensive but also cause environmental pollution. Our method does not require the use of any acid and methanol nor tricarboxylic acid. With the minimal pollution produced, it can be considered as more environment friendly than the other protein-staining methods [15]–[17]. The protocol includes only staining and quick washing steps, whereas traditional post-electrophoretic process is time consuming and cumbersome because it involves fixing, staining of protein gels, and destaining, and requires several hours to several days to finish the whole staining process [19]–[21]. The protocol described in the present study is faster than colloidal staining and other fast staining procedures [15], [17]. The whole post-electrophoretic process can be finished within 30 min. Thus, it is more time-saving compared with any other protein-staining methods. Despite the advantages of simplicity, time-saving ability, and environmental friendliness, several disadvantages exist, such as the need to boil the staining solution, unclear background, etc. The protocol requires keeping staining solution at a boiling temperature. Therefore, we suggest boiling the solution at a lower temperature to avoid breakage of the sheet. Increasing the purity or lengthening the destaining time from 10 to 12 h can produce a clearer background. The addition of 10% acetic acid to the destaining solution can also help in achieving better destaining effects.
